# [(3a*S*,5a*R*,8a*R*,8b*S*)-2,2,7,7-Tetra­methyl­tetra­hydro-3a*H*-bis­[1,3]dioxolo[4,5-*b*:4′,5′-*d*]pyran-3a-yl]methyl (*R*)-*N*-(1-phenyl­eth­yl)sulfamate

**DOI:** 10.1107/S1600536812016704

**Published:** 2012-04-28

**Authors:** Meng Xie, Si-Si Shen, Bao-Feng Chen, Yu Sha

**Affiliations:** a School of Pharmaceutical Engineering, Shenyang Pharmaceutical University, Mail Box 40, 103 Wenhua Road, Shenhe District, Shenyang 110016, People’s Republic of China

## Abstract

In the title compound, C_20_H_29_NO_8_S, the two five-membered rings adopt envelope conformations (with an O atom at the flap in each case), while the six-membered pyran ring displays a twist-boat conformation. In the crystal, mol­ecules are linked by N—H⋯O hydrogen bonds into a supra­molecular chain running along the *a* axis.

## Related literature

For general background to the drug topiramate [systematic name: 2,3:4,5-bis-*O*-(1-methyl­ethyl­idene)-beta-d-fructopyran­ose sulfamate] and its potential bioactivity, see: Maryanoff (2009 ▶); Maryanoff *et al.* (2008 ▶). For related structures, see: Maryanoff *et al.* (1998 ▶); Winum *et al.* (2006 ▶).
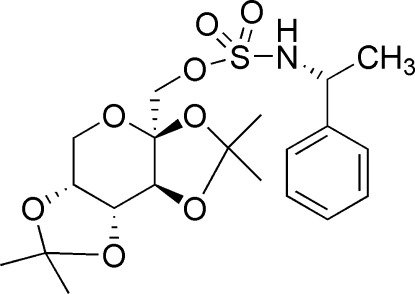


## Experimental

### 

#### Crystal data

C_20_H_29_NO_8_S
*M* *_r_* = 443.50
Orthorhombic, 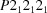

*a* = 9.5733 (9) Å
*b* = 15.0134 (14) Å
*c* = 15.9462 (15) Å
*V* = 2291.9 (4) Å^3^
*Z* = 4
Mo *K*α radiation
μ = 0.19 mm^−1^
*T* = 293 K
0.28 × 0.20 × 0.15 mm

#### Data collection

Bruker APEX CCD area-detector diffractometer
Absorption correction: multi-scan (*SADABS*; Sheldrick, 1996 ▶) *T*_min_ = 0.950, *T*_max_ = 0.973
13609 measured reflections
4041 independent reflections
3348 reflections with *I* > 2σ(*I*)
*R*_int_ = 0.029

#### Refinement

*R*[*F* ^2^ > 2σ(*F* ^2^)] = 0.041
*wR*(*F* ^2^) = 0.100
*S* = 1.04
4041 reflections
276 parameters
H-atom parameters constrained
Δρ_max_ = 0.14 e Å^−3^
Δρ_min_ = −0.19 e Å^−3^
Absolute structure: Flack (1983 ▶), 1736 Friedel pairs
Flack parameter: −0.02 (8)

### 

Data collection: *SMART* (Bruker, 2007 ▶); cell refinement: *SAINT* (Bruker, 2007 ▶); data reduction: *SAINT*; program(s) used to solve structure: *SHELXTL* (Sheldrick, 2008 ▶); program(s) used to refine structure: *SHELXTL*; molecular graphics: *SHELXTL*; software used to prepare material for publication: *SHELXTL*.

## Supplementary Material

Crystal structure: contains datablock(s) I, global. DOI: 10.1107/S1600536812016704/xu5509sup1.cif

Structure factors: contains datablock(s) I. DOI: 10.1107/S1600536812016704/xu5509Isup2.hkl

Additional supplementary materials:  crystallographic information; 3D view; checkCIF report

## Figures and Tables

**Table 1:** Hydrogen-bond geometry (Å, °)

*D*—H⋯*A*	*D*—H	H⋯*A*	*D*⋯*A*	*D*—H⋯*A*
N1—H1*A*⋯O3^i^	0.93	2.06	2.959 (3)	163

Symmetry code: (i) 
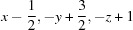
.

**Fig. 1.**
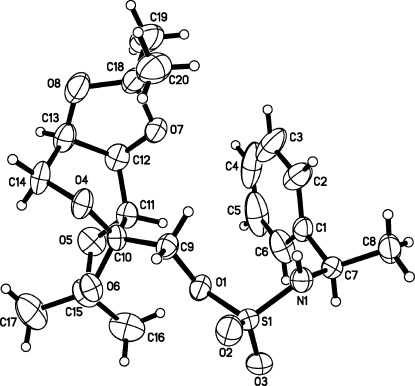
The structure of the title compound(I), showing 30% probability displacement ellipsoids and the atom-numbering scheme.

**Fig. 2.**
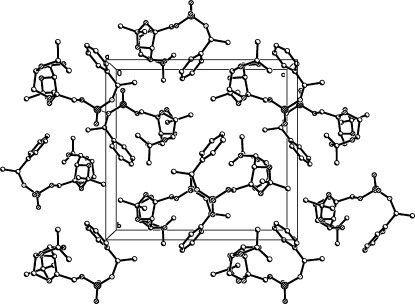
The molecular packing of (I), the molecular packing viewed along the b axis, H atoms not involved in hydrogen bonding have been omitted.

## supplementary crystallographic information

### Comment

The marketed drug Topiramate is a moderate inhibitor of carbonic anhydrase-II(CA—II), which is marketed worldwide for the treatmen of epilepsy and the prophylaxis of migraine headache(Maryanoff *et al.*, 2008). Topiramate easily qualifies as a "billion-dollar molecule". Given the disabling effects of epileptic seizures and the misery associated with recurrent migraine attacks, this drug has helped millions of patients in need across the globe (Maryanoff, 2009). Topiramate is a sulfa-derivative monosaccharide, it contains a sulfamide group, but a sulfamate group is much more effective than the sulfamide group for inhibiting human CA—II (Maryanoff *et al.*, 1998; Winum *et al.*, 2006).

As the derivative of Topiramate that contains a sulfamate group are of great pharmaceutical importance, we have undertaken the crystal structure determination of the title compound. C_20_H_29_N_2_O_8_S, by X-ray diffraction in the crystal packing of (I),[Fig. 2] The bond lengths and angles are within normal ranges. The six-membered ring O4—C10—C11—C12—C13—C14 (substituted pyranoid) is far from planar, and its shape approximates to a twist-boat conformation. In this description applied to this title compound (Fig. 1), atoms C10, C11, C13 and C14 from the bottom of the boat (deviation from the mean C10/C11/C13/C14 plane = 0.176 (4) Å), O4 the prow, and C12 the stern [deviations from the C10/C11/C13/C14 mean plane = 0.557, 0.387 Å, respectively]. The two five-membered rings C10—O6—C15—O5—C11 and C12—O7—C18—O8—C13 (substituted 1,3-dioxolane rings) are non-planar and adopt nearly envelope conformation (deviation from the mean C10/O6/C15/ C11 plane = 0.007 (4) Å, C12/ C18/O8/C13 plane= 0.038 (4) Å). The O5 atom is located above the plane [deviations from the C10/N2/C13/C12 mean plane = 0.473 Å], while the O7 atom is located above the plane [deviations from the C12/ C13/ O8/C18 mean plane = 0.363 Å], Atom C7 of the title molecule is chiral: *R* configuration was assigned to this atom based on the known chirality of the equivalent atom in the starting material, otherwise, the C10, C11, C12, C13 of the title molecule is chiral: S, S, *R*, *R*, respectively. The suIfonyl O atom is engaged in H-bonding with the N—H of the adjacent molecular.

### Experimental

((3aS,5aR,8aR,8bS)-2,2,7,7-tetramethyltetrahydro-3aH-bis[1,3]dioxolo [4,5 - b:4',5'-d]pyran-3a-yl)methyl sulfochloridate(7.16 g, 20 mmol) was placed in a round-bottomed flask, and dissolved in acetone(100 ml). Triethylamine(100 mmol)was added and then the (*s*)-benzenemethanamin (2.57 ml, 20 mmol) and the solution was heated to reflux for 2 h. The mixture was filtered and the filtrate was concentrated under vacuum. The pure product was obtained through silica gel chromatography(eluant petroleum ether:ethyl acetate, 2:1). Single crystals suitable for X-ray diffraction were prepared by slow evaporation of a dilute solution of the title compound in n-hexane:ethyl acetate,6:1 at room temperature.

### Refinement

The H atom of the NH group was located in a difference map, other H atoms were positioned with idealized geometry using a riding model with C—H = 0.93–0.98 Å. H atoms were refined in riding mode with *U*_iso_(H) = 1.2*U*_eq_(parent atom).

### Crystal data

**Table:**

C_20_H_29_NO_8_S	*D*_x_ = 1.285 Mg m^−^^3^
*M**_r_* = 443.50	Mo *K*α radiation, λ = 0.71073 Å
Orthorhombic, *P*2_1_2_1_2_1_	Cell parameters from 3967 reflections
*a* = 9.5733 (9) Å	θ = 2.5–20.8°
*b* = 15.0134 (14) Å	µ = 0.19 mm^−^^1^
*c* = 15.9462 (15) Å	*T* = 293 K
*V* = 2291.9 (4) Å^3^	Block, colourless
*Z* = 4	0.28 × 0.20 × 0.15 mm
*F*(000) = 944	

### Data collection

**Table:**

Bruker APEX CCD area-detector diffractometer	4041 independent reflections
Radiation source: fine-focus sealed tube	3348 reflections with *I* > 2σ(*I*)
Graphite monochromator	*R*_int_ = 0.029
φ and ω scans	θ_max_ = 25.0°, θ_min_ = 1.9°
Absorption correction: multi-scan (*SADABS*; Sheldrick, 1996)	*h* = −11→11
*T*_min_ = 0.950, *T*_max_ = 0.973	*k* = −17→16
13609 measured reflections	*l* = −18→18

### Refinement

**Table:**

Refinement on *F*^2^	Secondary atom site location: difference Fourier map
Least-squares matrix: full	Hydrogen site location: inferred from neighbouring sites
*R*[*F*^2^ > 2σ(*F*^2^)] = 0.041	H-atom parameters constrained
*wR*(*F*^2^) = 0.100	*w* = 1/[σ^2^(*F*_o_^2^) + (0.0565*P*)^2^ + 0.0232*P*] where *P* = (*F*_o_^2^ + 2*F*_c_^2^)/3
*S* = 1.04	(Δ/σ)_max_ = 0.001
4041 reflections	Δρ_max_ = 0.14 e Å^−^^3^
276 parameters	Δρ_min_ = −0.19 e Å^−^^3^
0 restraints	Absolute structure: Flack (1983), 1736 Friedel pairs
Primary atom site location: structure-invariant direct methods	Flack parameter: −0.02 (8)

### Special details

**Table:**

Geometry. All e.s.d.'s (except the e.s.d. in the dihedral angle between two l.s. planes) are estimated using the full covariance matrix. The cell e.s.d.'s are taken into account individually in the estimation of e.s.d.'s in distances, angles and torsion angles; correlations between e.s.d.'s in cell parameters are only used when they are defined by crystal symmetry. An approximate (isotropic) treatment of cell e.s.d.'s is used for estimating e.s.d.'s involving l.s. planes.
Refinement. Refinement of *F*^2^ against ALL reflections. The weighted *R*-factor *wR* and goodness of fit *S* are based on *F*^2^, conventional *R*-factors *R* are based on *F*, with *F* set to zero for negative *F*^2^. The threshold expression of *F*^2^ > σ(*F*^2^) is used only for calculating *R*-factors(gt) *etc*. and is not relevant to the choice of reflections for refinement. *R*-factors based on *F*^2^ are statistically about twice as large as those based on *F*, and *R*- factors based on ALL data will be even larger.

### Fractional atomic coordinates and isotropic or equivalent isotropic displacement parameters (Å^2^)

**Table:**

	*x*	*y*	*z*	*U*_iso_*/*U*_eq_	
S1	0.19640 (7)	0.77181 (4)	0.55630 (4)	0.05958 (19)	
O1	0.15464 (15)	0.71781 (12)	0.63697 (9)	0.0589 (4)	
O2	0.1225 (3)	0.85374 (12)	0.55749 (13)	0.0841 (6)	
O3	0.34363 (19)	0.76955 (16)	0.55879 (13)	0.0908 (7)	
O4	−0.12573 (18)	0.71591 (11)	0.78266 (10)	0.0623 (5)	
O5	0.1236 (2)	0.59605 (12)	0.84887 (12)	0.0785 (6)	
O6	0.11065 (19)	0.73889 (11)	0.80456 (10)	0.0623 (5)	
O7	−0.15017 (18)	0.53273 (13)	0.70728 (12)	0.0706 (5)	
O8	−0.3187 (2)	0.55560 (17)	0.80406 (14)	0.0970 (7)	
N1	0.1465 (2)	0.71749 (13)	0.47609 (12)	0.0551 (5)	
H1A	0.0538	0.7337	0.4685	0.066*	
C1	0.1733 (3)	0.55498 (16)	0.50384 (15)	0.0590 (6)	
C2	0.0399 (3)	0.5210 (2)	0.5013 (2)	0.0891 (10)	
H2	−0.0258	0.5453	0.4650	0.107*	
C3	0.0038 (5)	0.4506 (3)	0.5529 (3)	0.1323 (19)	
H3	−0.0864	0.4278	0.5510	0.159*	
C4	0.0982 (9)	0.4147 (3)	0.6059 (3)	0.155 (3)	
H4	0.0730	0.3675	0.6405	0.186*	
C5	0.2294 (8)	0.4476 (3)	0.6085 (3)	0.140 (2)	
H5	0.2944	0.4231	0.6452	0.168*	
C6	0.2678 (4)	0.5177 (2)	0.5567 (2)	0.0941 (11)	
H6	0.3589	0.5392	0.5582	0.113*	
C7	0.2137 (2)	0.63303 (15)	0.44935 (14)	0.0525 (5)	
H7	0.3150	0.6409	0.4541	0.063*	
C8	0.1798 (3)	0.6189 (2)	0.35758 (16)	0.0782 (8)	
H8A	0.0805	0.6136	0.3508	0.117*	
H8B	0.2242	0.5654	0.3382	0.117*	
H8C	0.2131	0.6687	0.3256	0.117*	
C9	0.0129 (2)	0.72335 (19)	0.66670 (14)	0.0575 (6)	
H9A	−0.0473	0.6859	0.6328	0.069*	
H9B	−0.0204	0.7842	0.6629	0.069*	
C10	0.0103 (3)	0.69241 (16)	0.75731 (14)	0.0512 (6)	
C11	0.0462 (3)	0.59463 (16)	0.77320 (16)	0.0594 (6)	
H11	0.1045	0.5717	0.7275	0.071*	
C12	−0.0800 (3)	0.53558 (18)	0.78492 (18)	0.0679 (7)	
H12	−0.0520	0.4757	0.8026	0.082*	
C13	−0.1909 (3)	0.57379 (19)	0.84437 (17)	0.0749 (8)	
H13	−0.1873	0.5424	0.8982	0.090*	
C14	−0.1693 (3)	0.67232 (19)	0.85826 (17)	0.0769 (9)	
H14A	−0.2558	0.6988	0.8778	0.092*	
H14B	−0.0990	0.6811	0.9013	0.092*	
C15	0.2006 (4)	0.67763 (19)	0.84864 (18)	0.0754 (8)	
C16	0.3370 (3)	0.6682 (2)	0.8038 (3)	0.1010 (12)	
H16A	0.3960	0.6276	0.8339	0.151*	
H16B	0.3818	0.7253	0.8003	0.151*	
H16C	0.3208	0.6457	0.7483	0.151*	
C17	0.2157 (5)	0.7101 (3)	0.9384 (2)	0.1217 (15)	
H17A	0.1248	0.7176	0.9628	0.183*	
H17B	0.2642	0.7661	0.9388	0.183*	
H17C	0.2677	0.6672	0.9703	0.183*	
C18	−0.2960 (3)	0.5233 (2)	0.72210 (19)	0.0735 (8)	
C19	−0.3362 (4)	0.4256 (3)	0.7196 (3)	0.1220 (14)	
H19A	−0.2824	0.3934	0.7603	0.183*	
H19B	−0.3178	0.4022	0.6647	0.183*	
H19C	−0.4338	0.4196	0.7321	0.183*	
C20	−0.3716 (4)	0.5806 (3)	0.6615 (2)	0.1097 (12)	
H20A	−0.4703	0.5760	0.6712	0.164*	
H20B	−0.3508	0.5616	0.6054	0.164*	
H20C	−0.3426	0.6414	0.6686	0.164*	

### Atomic displacement parameters (Å^2^)

**Table:**

	*U*^11^	*U*^22^	*U*^33^	*U*^12^	*U*^13^	*U*^23^
S1	0.0639 (4)	0.0605 (4)	0.0542 (3)	−0.0178 (3)	0.0103 (3)	−0.0063 (3)
O1	0.0513 (9)	0.0788 (11)	0.0467 (9)	0.0067 (8)	0.0047 (7)	0.0040 (8)
O2	0.1259 (17)	0.0482 (10)	0.0781 (13)	−0.0087 (10)	0.0276 (13)	−0.0058 (10)
O3	0.0632 (12)	0.1228 (17)	0.0865 (13)	−0.0377 (11)	0.0139 (10)	−0.0305 (14)
O4	0.0721 (10)	0.0594 (10)	0.0555 (10)	0.0228 (8)	0.0178 (8)	0.0063 (9)
O5	0.0932 (14)	0.0661 (12)	0.0762 (13)	0.0151 (10)	−0.0197 (11)	0.0193 (10)
O6	0.0838 (11)	0.0538 (10)	0.0495 (9)	0.0132 (9)	−0.0116 (8)	−0.0001 (8)
O7	0.0681 (12)	0.0765 (12)	0.0671 (12)	−0.0053 (9)	0.0197 (10)	−0.0090 (10)
O8	0.0785 (14)	0.1293 (19)	0.0832 (14)	0.0074 (13)	0.0282 (12)	−0.0074 (14)
N1	0.0599 (12)	0.0553 (12)	0.0501 (11)	0.0039 (10)	−0.0009 (9)	0.0037 (9)
C1	0.0752 (18)	0.0509 (13)	0.0508 (14)	0.0014 (13)	0.0123 (12)	−0.0048 (11)
C2	0.091 (2)	0.0660 (19)	0.111 (3)	−0.0071 (16)	0.040 (2)	0.0001 (19)
C3	0.172 (4)	0.067 (2)	0.158 (4)	−0.026 (3)	0.110 (4)	−0.011 (3)
C4	0.327 (9)	0.051 (2)	0.086 (3)	0.015 (4)	0.099 (5)	0.006 (2)
C5	0.284 (8)	0.072 (3)	0.063 (2)	0.047 (4)	0.000 (4)	0.005 (2)
C6	0.146 (3)	0.0672 (19)	0.0687 (19)	0.0207 (19)	−0.022 (2)	−0.0035 (17)
C7	0.0501 (13)	0.0562 (13)	0.0511 (13)	−0.0018 (10)	0.0042 (12)	−0.0031 (12)
C8	0.103 (2)	0.0783 (18)	0.0530 (15)	−0.0034 (17)	0.0046 (16)	−0.0068 (14)
C9	0.0544 (14)	0.0732 (17)	0.0449 (12)	0.0045 (13)	0.0020 (10)	0.0071 (13)
C10	0.0605 (14)	0.0505 (13)	0.0426 (12)	0.0122 (11)	0.0030 (11)	−0.0020 (10)
C11	0.0684 (16)	0.0535 (14)	0.0562 (15)	0.0158 (12)	0.0046 (13)	0.0027 (12)
C12	0.0800 (19)	0.0528 (15)	0.0709 (18)	0.0119 (14)	0.0089 (15)	0.0088 (14)
C13	0.091 (2)	0.0798 (19)	0.0543 (15)	0.0030 (17)	0.0198 (16)	0.0136 (14)
C14	0.095 (2)	0.087 (2)	0.0487 (15)	0.0202 (17)	0.0263 (15)	0.0026 (14)
C15	0.095 (2)	0.0631 (17)	0.0684 (18)	0.0118 (16)	−0.0278 (17)	0.0084 (14)
C16	0.075 (2)	0.087 (2)	0.141 (3)	0.0195 (17)	−0.036 (2)	0.010 (2)
C17	0.176 (4)	0.121 (3)	0.068 (2)	−0.001 (3)	−0.055 (2)	0.009 (2)
C18	0.0679 (18)	0.0804 (18)	0.0722 (18)	−0.0053 (15)	0.0199 (16)	0.0013 (16)
C19	0.117 (3)	0.091 (2)	0.158 (4)	−0.032 (2)	0.040 (3)	−0.005 (3)
C20	0.085 (2)	0.138 (3)	0.106 (3)	−0.005 (2)	−0.008 (2)	0.027 (3)

### Geometric parameters (Å, º)

**Table:**

S1—O3	1.411 (2)	C8—H8A	0.9600
S1—O2	1.419 (2)	C8—H8B	0.9600
S1—O1	1.5722 (17)	C8—H8C	0.9600
S1—N1	1.590 (2)	C9—C10	1.518 (3)
O1—C9	1.440 (3)	C9—H9A	0.9700
O4—C10	1.409 (3)	C9—H9B	0.9700
O4—C14	1.434 (3)	C10—C11	1.529 (3)
O5—C11	1.416 (3)	C11—C12	1.510 (4)
O5—C15	1.430 (3)	C11—H11	0.9800
O6—C10	1.406 (3)	C12—C13	1.535 (4)
O6—C15	1.443 (3)	C12—H12	0.9800
O7—C12	1.409 (3)	C13—C14	1.510 (4)
O7—C18	1.423 (3)	C13—H13	0.9800
O8—C13	1.408 (4)	C14—H14A	0.9700
O8—C18	1.411 (3)	C14—H14B	0.9700
N1—C7	1.485 (3)	C15—C16	1.496 (5)
N1—H1A	0.9275	C15—C17	1.519 (4)
C1—C6	1.358 (4)	C16—H16A	0.9600
C1—C2	1.375 (4)	C16—H16B	0.9600
C1—C7	1.509 (3)	C16—H16C	0.9600
C2—C3	1.383 (5)	C17—H17A	0.9600
C2—H2	0.9300	C17—H17B	0.9600
C3—C4	1.349 (8)	C17—H17C	0.9600
C3—H3	0.9300	C18—C20	1.483 (4)
C4—C5	1.350 (8)	C18—C19	1.516 (5)
C4—H4	0.9300	C19—H19A	0.9600
C5—C6	1.387 (6)	C19—H19B	0.9600
C5—H5	0.9300	C19—H19C	0.9600
C6—H6	0.9300	C20—H20A	0.9600
C7—C8	1.514 (4)	C20—H20B	0.9600
C7—H7	0.9800	C20—H20C	0.9600
			
O3—S1—O2	121.23 (14)	C12—C11—C10	113.8 (2)
O3—S1—O1	102.63 (12)	O5—C11—H11	110.0
O2—S1—O1	108.03 (11)	C12—C11—H11	110.0
O3—S1—N1	108.09 (11)	C10—C11—H11	110.0
O2—S1—N1	107.80 (13)	O7—C12—C11	106.9 (2)
O1—S1—N1	108.49 (10)	O7—C12—C13	103.0 (2)
C9—O1—S1	118.64 (14)	C11—C12—C13	114.3 (2)
C10—O4—C14	113.32 (19)	O7—C12—H12	110.8
C11—O5—C15	106.29 (19)	C11—C12—H12	110.8
C10—O6—C15	110.64 (19)	C13—C12—H12	110.8
C12—O7—C18	108.9 (2)	O8—C13—C14	112.1 (3)
C13—O8—C18	110.8 (2)	O8—C13—C12	104.3 (2)
C7—N1—S1	122.60 (16)	C14—C13—C12	111.2 (2)
C7—N1—H1A	126.9	O8—C13—H13	109.7
S1—N1—H1A	104.9	C14—C13—H13	109.7
C6—C1—C2	118.9 (3)	C12—C13—H13	109.7
C6—C1—C7	120.4 (3)	O4—C14—C13	111.3 (2)
C2—C1—C7	120.6 (3)	O4—C14—H14A	109.4
C1—C2—C3	119.9 (4)	C13—C14—H14A	109.4
C1—C2—H2	120.1	O4—C14—H14B	109.4
C3—C2—H2	120.1	C13—C14—H14B	109.4
C4—C3—C2	120.7 (5)	H14A—C14—H14B	108.0
C4—C3—H3	119.7	O5—C15—O6	103.9 (2)
C2—C3—H3	119.7	O5—C15—C16	111.8 (3)
C5—C4—C3	119.8 (5)	O6—C15—C16	110.4 (2)
C5—C4—H4	120.1	O5—C15—C17	108.8 (3)
C3—C4—H4	120.1	O6—C15—C17	108.1 (3)
C4—C5—C6	120.3 (5)	C16—C15—C17	113.4 (3)
C4—C5—H5	119.8	C15—C16—H16A	109.5
C6—C5—H5	119.8	C15—C16—H16B	109.5
C1—C6—C5	120.4 (4)	H16A—C16—H16B	109.5
C1—C6—H6	119.8	C15—C16—H16C	109.5
C5—C6—H6	119.8	H16A—C16—H16C	109.5
N1—C7—C1	112.74 (18)	H16B—C16—H16C	109.5
N1—C7—C8	107.7 (2)	C15—C17—H17A	109.5
C1—C7—C8	113.1 (2)	C15—C17—H17B	109.5
N1—C7—H7	107.7	H17A—C17—H17B	109.5
C1—C7—H7	107.7	C15—C17—H17C	109.5
C8—C7—H7	107.7	H17A—C17—H17C	109.5
C7—C8—H8A	109.5	H17B—C17—H17C	109.5
C7—C8—H8B	109.5	O8—C18—O7	105.7 (2)
H8A—C8—H8B	109.5	O8—C18—C20	109.2 (3)
C7—C8—H8C	109.5	O7—C18—C20	108.2 (2)
H8A—C8—H8C	109.5	O8—C18—C19	108.6 (3)
H8B—C8—H8C	109.5	O7—C18—C19	109.9 (3)
O1—C9—C10	108.12 (18)	C20—C18—C19	114.8 (3)
O1—C9—H9A	110.1	C18—C19—H19A	109.5
C10—C9—H9A	110.1	C18—C19—H19B	109.5
O1—C9—H9B	110.1	H19A—C19—H19B	109.5
C10—C9—H9B	110.1	C18—C19—H19C	109.5
H9A—C9—H9B	108.4	H19A—C19—H19C	109.5
O6—C10—O4	110.70 (18)	H19B—C19—H19C	109.5
O6—C10—C9	110.3 (2)	C18—C20—H20A	109.5
O4—C10—C9	102.21 (18)	C18—C20—H20B	109.5
O6—C10—C11	103.55 (19)	H20A—C20—H20B	109.5
O4—C10—C11	113.6 (2)	C18—C20—H20C	109.5
C9—C10—C11	116.6 (2)	H20A—C20—H20C	109.5
O5—C11—C12	108.8 (2)	H20B—C20—H20C	109.5
O5—C11—C10	104.1 (2)		

### Hydrogen-bond geometry (Å, º)

**Table:**

*D*—H···*A*	*D*—H	H···*A*	*D*···*A*	*D*—H···*A*
N1—H1*A*···O3^i^	0.93	2.06	2.959 (3)	163

Symmetry code: (i) *x*−1/2, −*y*+3/2, −*z*+1.
